# No Relation between Psoriasis and Renal Abnormalities: A Case-Control Study

**DOI:** 10.1155/2018/5301631

**Published:** 2018-02-11

**Authors:** Zohreh Tehranchinia, Esmat Ghanei, Nahid Mohammadi, Masoud Partovi-Kia, Hoda Rahimi, Nikoo Mozafari

**Affiliations:** ^1^Skin Research Center, Shahid Beheshti University of Medical Sciences, Tehran, Iran; ^2^Internal Medicines Ward, Shahid Beheshti University of Medical Sciences, Tehran, Iran

## Abstract

Multiple observational studies have demonstrated that psoriasis is associated with nephropathy; however, the renal involvement in psoriasis remains largely a matter of debate. The current study was designed to investigate if psoriatic patients are at increased risk of renal abnormalities, in absence of any other comorbidities. Forty patients (11 women, 29 men, mean age 44.9 ± 15.45 years) with moderate to severe chronic plaque type psoriasis who were not under systemic therapy and 40 age- and gender-matched control subjects were enrolled in the study. Patients and controls with history of diabetes, hypertension, and chronic renal disease were excluded. Urinalysis by dipstick and microscopic evaluation and 24 h proteinuria and albuminuria were measured in all patients and controls. Patients with psoriasis and controls were not significantly different with respect to the prevalence of abnormal urinalysis (7.5% versus 5%,* P* = 1.0), mean 24 h proteinuria (70.40 ± 24.38 mg/24 h versus 89.40 ± 26.78 mg/24 h,* P* = 0.30), and albuminuria (14.15 ± 8.12 mg/24 h versus 16.62 ± 8.21 mg/24 h,* P* = 0.18). The presence of abnormal urinalysis was not more common in patients with psoriasis than in controls. Our study demonstrated that psoriatic patients without any other comorbidities are not at increased risk of kidney disease.

## 1. Introduction

Psoriasis is a common chronic inflammatory disease affecting 2-3% of the world's population which is characterized by the development of inflammatory plaques on the skin [1]. Recent evidence suggests that psoriasis is not a disease limited to the skin; it has far-reaching systemic effects [2].

Multiple observational studies have demonstrated that psoriasis is associated with comorbidities such as arthritis, diabetes, cardiovascular disease, chronic obstructive pulmonary disease, and hypertension [2–4].

Based on recently published reports, an association between psoriasis and nephropathy has been proposed; however, the renal involvement in psoriasis remains largely a matter of debate [5, 6].

Multiple cross-sectional studies have shown greater prevalence of microalbuminuria, a sign of subclinical glomerular dysfunction in patients with psoriasis [7, 8]. Yet other studies have detected no association between psoriasis and renal disease [9–11].

There are some potential confounders such as diabetes, hypertension, and use of nephrotoxic drugs. It is not totally clear whether observed nephropathy is caused by psoriasis or is the result of drugs used for psoriasis treatment or is secondary to comorbidities associated with psoriasis [5, 6]. Furthermore, psoriatic arthritis and consuming NSAIDs as potential cofounder may increase the risk of renal abnormality in psoriatic patients [12].

The current study was designed to investigate if psoriatic patients are at increased risk of renal abnormalities, in absence of any other comorbidities.

## 2. Material and Methods

Forty patients with moderate to severe chronic plaque type psoriasis who were not under systemic therapy were recruited. Forty healthy control subjects frequently matched for age and sex, who visited our outpatient clinic for excision of benign lesions, were included in this study. Exclusion criteria for patients and controls were as follows: (1) known cases of hypertension and diabetes; (2) subjects with obvious cause of chronic renal failure or glomerulonephritis; (3) receiving prior systemic treatment (including cyclosporine, methotrexate, acitretin, TNF-alpha inhibitors, and NSAIDs); (4) pregnant or lactating subjects; and (5) patients with psoriatic arthritis.

The study design and protocol were approved by the ethics committee of our skin research center and informed consent was obtained from all participants.

Baseline demographic data of all subjects were recorded at the start of the study. For assessment of disease severity in psoriatic patients we used psoriasis area severity index (PASI) scoring. Based on recent guidelines, moderate to severe psoriasis is defined as PASI score > 10 [2].

Five milliliters of venous blood was collected from each subject for the determination of serum sodium (Na), potassium (K), creatinine (Cr), and blood nitrogen urea (BUN).

For measuring creatinine clearance and quantifying total proteinuria and albuminuria, all study participants were asked to collect 24 h urine specimens (from 8 a.m. to 8 a.m. the following day, according to standard protocol). Random midstream urine was collected from each subject for urinalysis. Samples were tested by dipstick and microscopy for the presence of RBC, protein, and other abnormalities.

Creatinine clearance was calculated using the following formula: urine creatinine/serum creatinine multiplied by 24 h urine volume (UCr/PCr) ×* V*. This was divided by 1440 to get the value in ml/min. Normal renal function was defined as a creatinine clearance of more than 60 mL/min.

Pathologic proteinuria was considered present if the dipstick reading was ≥1+ or if the total protein excretion was more than 150 mg/24 h. Pathologic albuminuria was defined as albumin excretion of more than 30 mg/24 h.

Microscopic hematuria was defined as ≥5 erythrocytes/HPF (high-power field). All abnormal urinalyses were performed twice.

## 3. Statistical Methods

Continuous variables are expressed as mean ± standard deviation (SD) unless otherwise stated. Categorical data are presented as number (percentage). The Shapiro-Wilk *W*-test was applied for examining the normality assumption of continuous variables.

For the continuous variables with skewed distributions and for normally distributed variables, nonparametric Mann–Whitney *U* test and Student's* t*-test were applied to compare the laboratory findings of patients with psoriasis and healthy controls.

Pearson's Chi-square test was used whenever the expected cell frequencies were at least 5. With small expected frequencies, Fisher's exact test was employed. Data analyses were performed using the statistical software SPSS 18.0.0. (SPSS Inc., Chicago, IL, USA). Two-sided *P* values less than 0.05 were considered statistically significant.

## 4. Results

Forty patients with psoriasis and forty age and sex frequency-matched healthy controls were enrolled in this study. The baseline demographics and clinical characteristics of the study participants are summarized in Table 1. Subjects in both groups were similar in age, gender, BMI, and diastolic and systolic blood pressure levels (Table 1).

The median serum levels of BUN, serum creatinine, sodium and potassium, creatinine clearance, and urinary protein and albumin excretion were not significantly different between patients and healthy controls (Table 2).

Of the patients with psoriasis, 3 (7.5%) had abnormal findings on dipstick and microscopic examination of the urine. Two had microscopic hematuria and one had +1 proteinuria. In the controls 2 (5%) had microscopic hematuria. Further testing identified that nephrolithiasis was the cause of hematuria in patients and controls. And subsequent quantitative assay in patient with proteinuria showed that the patient had proteinuria less than 150 mg/24 h.

## 5. Discussion

The present study was designed to evaluate the presence of renal abnormalities in moderately to severely affected patients with psoriasis. Our results did not show an increased risk of renal abnormality in psoriatic patients. The urine analysis by dipstick and microscopic evaluation was similar in psoriatic patients and controls. Urine albumin or protein excretion was not significantly different between two groups.

Recently, psoriasis has been considered to be a systemic disorder, affecting organs beyond the skin [3]. Increased incidence of nephropathy in psoriatic patients has been suggested by several studies and the new concept of “psoriatic nephropathy” has also been proposed, but the link between the two diseases is still controversial [5, 6].

The hypothesis of psoriatic nephropathy has been generated based on case reports of glomerulonephritis in patients with psoriasis and supported by cross-sectional and observational epidemiologic studies [5, 7, 8, 12, 13].

Microalbuminuria is considered to be a marker of glomerular damage and can be used to detect glomerular damage at the reversible stage. Some studies performed in patients with psoriasis have found increased urinary albumin in psoriatic patients compared with healthy controls [7, 8].

In a recent cross-sectional study, Khan and colleagues demonstrated a greater incidence of proteinuria and elevated serum creatinine and urea levels in patients suffering from psoriasis [13]. They also showed renal impairment is more prevalent in psoriatic patients especially in subjects with concomitant psoriatic arthritis [13].

In 2013 using a United Kingdom primary care electronic medical record database, Wan et al. identified 143,883 patients with psoriasis and matched them with up to five controls without psoriasis each. The researchers found that patients with psoriasis, particularly those with severe disease were at nearly doubled risk of developing moderate to advanced chronic kidney disease compared with control patients [5].

A similar finding emerged from Taiwan in 2015, using the national health care database; Chiu et al. followed 4344 patients with psoriasis and 13032 subjects as controls, for up to 5 years [12]. They found patients with psoriasis had an increased risk of developing glomerular nephritis and chronic kidney disease. The risk increased among patients with more severe disease, patients with arthritis, and those taking NSAIDs [12], while in this study severe psoriasis was defined as if patients received systemic therapies or phototherapy. This approach may affect the accuracy of study, as systemic treatment for psoriasis may itself increase the risk of developing renal comorbidity.

However contrary to the aforementioned studies multiple cross-sectional studies and one prevalence study failed to show an association between psoriasis and renal abnormalities [9–11, 14].

In an early study of renal function in psoriatic patients, Kaftan et al. were unable to find a significant difference in urinary albumin excretion or creatinine clearance between psoriatic patients and healthy subjects [11]. Dervisoglu et al. in a cross-sectional study on 45 patients with psoriasis showed no significant difference in urinary albumin excretion between psoriatic patients and healthy subjects [10].

In a retrospective chart review, between 1997 and 2000, Pearce et al. studied 753 patients from an academic dermatology practice to identify the presence of comorbidities in patients with psoriasis. A comorbid diagnosis was confirmed in 551 patients (73%), with hypertension, dyslipidemia, diabetes, and heart disease being the most and renal failure and hepatitis being the least common identified ones. When compared with national prevalence estimates, no increased rate of renal failure was observed among psoriasis patients [14].

The risk of systemic involvement increases with increasing disease severity among patients with psoriasis. In a study by Yeung et al. kidney diseases were more prevalent in patients with moderate to severe psoriasis than in those with mild disease [15].

Accordingly, in our study we include moderately to severely affected patients with psoriasis. Furthermore to eliminate potential confounders we also excluded patients with diabetes and hypertension and those who were taking NSAIDs or other potentially nephrotoxic drugs used for treatment of psoriasis.

Our data did not reveal any association between psoriasis and kidney disease such as microalbuminuria or glomerular nephritis. Our results are consistent with Cassano et al. who describe no abnormalities in renal function (mainly urinary albumin excretion and creatinine clearance) in psoriatic patients who had never been exposed to nephrotoxic drugs [9].

The mechanisms mediating renal insufficiency in psoriasis remains largely unclear [6]. There are three possibilities: (1) chronic inflammation driven by cell mediated immunity with T-cell activation and variety of interleukins and cytokines, including TNF*α*, is thought to be the cause of glomerular injury in psoriasis; (2) drugs used for psoriasis, in particular methotrexate and cyclosporine, could cause kidney damage; (3) given the higher prevalence of diabetes and hypertension among patients with psoriasis, observed renal abnormalities in psoriatic patients could be the consequence of these comorbidities [6].

In our study, the possible explanation of the untouched renal function might be the small number of available cases which precluded our ability to reach firm conclusion. But it is also possible that patients with only cutaneous form of disease whose systemic therapies have not been started yet are not at increased risk of renal impairments.

Another explanation is that the duration of the disease may have an effect on prevalence of comorbidities. Since in our study we included those who were all naïve to systemic antipsoriasis therapy, most of them were patients whose disease had been started or aggravated in recent one to two years. Despite the growing literature on comorbidity risks in psoriasis, there remains a critical knowledge gap on the degree to which psoriasis duration may affect the prevalence of major medical comorbidity, which needs to be addressed in future studies.

## 6. Conclusion

In summary our study demonstrated that moderately to severely affected psoriatic patients whose disease has been newly diagnosed or aggravated and who do not have any other comorbidities and are not taking NSAIDs or other nephrotoxic drugs are not at increased risk of kidney disease. However our results require confirmation in large patient populations in prospective studies.

## Figures and Tables

**Table 1:** Demographics and clinical features of patients with psoriasis and healthy controls.

Characteristic	Patients with psoriasis (*n* = 40)	Healthy controls (*n* = 40)	*P* value
Gender, number (%)			
Female	11 (28)	11 (28)	
Male	29 (72)	29 (72)	
Age, years (mean ± SD)	44.90 ± 15.45	44.92 ± 15.40	0.99
BMI	27.08 ± 4.47	24.17 ± 2.52	0.15
Systolic BP, mm Hg	118.87 ± 10.88	116.00 ± 11.04	0.24
Diastolic BP, mm Hg	68.87 ± 7.63	69.00 ± 9.07	0.94
PASI score (mean ± SD)	28.84 ± 12.48		

**Table 2:** Laboratory findings of patients with psoriasis and healthy controls.

Parameters	Patients with psoriasis (*n* = 40)	Healthy controls (*n* = 40)	*P* value
BUN, mg/dl	34.37 ± 8.32	32.85 ± 7.23	0.32
Creatinine, mg/dl			
Females	0.92 ± 0.19	0.88 ± 0.18	0.37
Males	1.05 ± 0.15	0.9 ± 0.20	0.50

Sodium, mEq/L	140.20 ± 3.40	139.80 ± 2.99	0.57
Potassium, mEq/L	4.17 ± 0.35	4.19 ± 0.37	0.80
24-hour urine volume, ml/24 h	1542.25 ± 544.33	1424.50 ± 565.37	0.42
24-hour urine protein, mg/24 h	70.40 ± 24.38	89.40 ± 26.78	0.30
24-hour urine creatinine, mg/24 h			
Females	1082.50 ± 365.33	1132.32 ± 381.12	0.58
Males	1286.22 ± 295.19	1331.65 ± 281.37	0.63
24-hour urine albumin, mg/24 h	14.15 ± 8.12	16.62 ± 8.21	0.18
Creatinine clearance, ml/min			
Females	89.26 ± 28.16	91.22 ± 24.36	0.63
Males	95.31 ± 24.92	96.72 ± 25.71	0.68
